# Enhancing High Reliability in Oncology Care: The Critical Role of Nurses—A Systematic Review and Thematic Analysis

**DOI:** 10.3390/healthcare13030283

**Published:** 2025-01-31

**Authors:** Hiroko Komatsu, Akemi Hara, Fumiko Koyama, Yasuhiro Komatsu

**Affiliations:** 1 Division of Faculty Development Nursing, Kindai University, 377-2 Ohnohigashi, Osakasayama 589-8511, Osaka, Japan; 2 Department of Healthcare Quality and Safety, Graduate School of Medicine, Gunma University, 3-39-22 Showa-machi, Maebashi 371-8511, Gunma, Japan

**Keywords:** high reliability, quality improvement, patient safety, oncology, nurse

## Abstract

**Background/objectives:** The oncology setting is complex and recognized as a high-risk area, with an increased potential for errors due to the complexity of therapeutic modalities and different processes. Nurses are pivotal in fostering a culture of patient safety and high reliability and actively contribute to the enhancement of safety standards in oncology care. This review systematically identified and examined the critical role of nurses in promoting high reliability within oncology organizations to ensure optimal patient outcomes. **Methods**: A systematic review was performed in the PubMed, CINAHL, and Cochran Library databases in accordance with PRISMA guidelines. Using Tomas and Harden’s three stages, 12 studies were deductively and thematically analyzed to discover themes. **Results:** We identified eight themes concerning nurses’ roles in achieving high reliability in healthcare within oncology organizations: establishing standardized and safe administration, enhancing situational awareness, promoting effective communication, advocating for patients, building a culture of safety, leading safety culture improvements, engaging staff, and fostering patient engagement. **Conclusions:** Nurses play a critical role in identifying, communicating, and correcting safety threats, overcoming various organizational barriers to safety concerns, and maintaining and developing a culture of safety for patients and families. Although this review included a relatively limited body of literature, the findings highlight the need for further research that considers the unique characteristics of health and healthcare systems. Protocol registry; UMIN Clinical Trials Registry (UMIN-CTR) Registry No. UMIN000056140.

## 1. Introduction

The incidence and mortality rates of cancer are increasing worldwide, posing significant global challenges [1]. Over the coming decades, cancer-related mortality is expected to rise, and in parallel with population projections, the burden on healthcare systems is anticipated to escalate [2]. Advances in cancer treatment have enabled high-risk patients, including the elderly and those with comorbidities, to receive therapies. However, this has also led to an increased risk of adverse events associated with cancer drug therapies. Therefore, establishing a safe and high-quality cancer treatment framework is an urgent priority.

Patient safety is a global public health concern, and the harm resulting from unsafe care is a significant and preventable cause of mortality and morbidity worldwide [3]. Causes of patient harm include medication errors, surgical errors, healthcare-associated infections, and diagnostic errors, with medication-related harm being the leading cause of patient harm [4]. The economic impact of medication-related harm is also substantial [5]. In 2017 and 2021, the WHO Health Organization designated medication safety as the focus of the Global Patient Safety Challenge, promoting worldwide efforts to enhance medication safety [6]. Chemotherapy plays a central role in cancer care, and strengthening the safety of cancer chemotherapy has emerged as a global priority [7].

Healthcare operates as a complex adaptive system comprising numerous inter-related components that interact dynamically [8,9]. Oncology, in particular, is recognized as a high-risk domain owing to the complexity of treatment modalities, multiple processes involved, and engagement of multidisciplinary teams, which together increase the likelihood of errors [10,11]. The complexity of cancer treatment is exacerbated by a delivery system that lacks integration and organization in its design [12]. Inadequate care coordination can result in poor symptom control, medical errors, and increased healthcare costs [13,14]. Even minor errors, if left unaddressed, can result in severe outcomes including mortality. Conversely, even significant errors may prevent serious long-term consequences when managed promptly and appropriately.

High-reliability organizations (HROs) are defined as those that operate in complex, high-hazard situations for extended periods while managing to avoid serious failures, such as nuclear power and aviation [15,16]. HROs provide highly reliable performance even in complex environments and serve as a model for enhancing safety in healthcare [17]. The WHO Global Patient Safety Action Plan regards building high-reliability systems as a key strategy, a principle also adopted by agencies such as AHRQ and the U.S. Department of Veterans Affairs [18,19,20,21]. A literature review has shown that HRO principles effectively promote patient safety and improve the quality of care [22]. Similarly, the role of nurses in strengthening quality and safety in oncology care is considered to align with HRO principles, emphasizing the need for a systematic review on this topic.

With an increasing number of high-risk patients undergoing interventions, ensuring high-quality care in a safer environment has become a challenging task, necessitating the establishment of highly reliable organizations. Nursing leaders actively promote the transition to patient safety and a high-reliability culture to enhance patient safety in oncology settings [23,24]. To establish a highly reliable organization, we emphasize the pivotal role of leadership among oncology nurses. The creation of mindful and resilient trust relationships within high-reliability institutions is expected to improve quality of life for both patients and healthcare providers. In this review, we explored the critical role of nurses in enhancing organization-wide patient safety and delivering high-quality care through the application of HRO principles in oncology, a field where errors and adverse events can have severe consequences for patients.

## 2. Methods

### 2.1. Approach

We conducted a systematic review and thematic analysis following the latest PRISMA guidelines to the extent possible [25]. The systematic review protocol was registered in UMIN Clinical Trials Registry (UMIN000056140 (https://center6.umin.ac.jp/cgi-open-bin/ctr/ctr_view.cgi?recptno=R000064146, accessed on 8 January 2025)). Given that PRISMA guidelines are primarily designed for quantitative systematic reviews and meta-analyses, we incorporated thematic analysis to address their limitations in synthesizing the qualitative literature. Our research question was the following: what role do nurses play in establishing high-reliability organizations that enhance organization-wide patient safety and quality within the field of cancer care?

### 2.2. Search Strategy

The search strategy was developed with an expert library and information specialist. A search of electronic databases—PubMed (via MEDLINE), the Cumulative Index to Nursing and Allied Health Literature (CINAHL), and the Cochrane Library (Trials only)—was conducted to identify relevant articles. Manual searches of reference lists and the gray literature were also performed. Searches were limited to articles published in English from database inception to February 2024. A broad range of key search terms using a combination of MeSH and free text keywords for “high-reliability organization”, “oncology”, and “nurses’ role” were utilized. Additional details on other MeSH terms and the combination of free-text searches can be found in Supplementary Files S1 and S2.

### 2.3. Eligibility Criteria

Studies were eligible for inclusion if they met the following criteria: (1) The study was either a qualitative, quantitative, mixed-method, or quality improvement study. (2) It described the critical role of nurses in establishing high-reliability organizations that enhance organization-wide patient safety and quality within the field of cancer care. (3) The study was published in a peer-reviewed journal from inception to February 2024. (4) The article was published in English. Reviews, letters, case studies, editorials, and conference abstracts were excluded. Two authors (H.K. and Y.K.) independently carried out these identifications, resolving any discrepancies through discussion.

### 2.4. Quality Appraisal

Since the methods used in the included studies were heterogeneous, different appraisal tools were used for each study type. The Quality Improvement Minimum Quality Criteria Set (QI-MQCS) was used to critically appraise the quality of the nine quality improvement studies included in this review [26]. This tool consists of 16 QI-MQCS criteria that assess the appropriateness and clarity of studies. The QI-MQCS enables reviewers to determine whether the 16 QI reporting standards have been “met”, “not met”, or “partially met”. The Mixed Methods Appraisal Tool (MMAT) was used to critically appraise the quality of the four studies that were not quality improvement studies [27]. The MMAT is designed for systematic mixed study reviews and includes qualitative, quantitative, and mixed-methods studies. It consists of five criteria assessing the appropriateness and clarity of studies, such as study aims and objectives, data collection methods, study findings, the interpretation of results, and coherence between data analysis and interpretation. Two authors (H.K. and Y.K.) independently carried out these appraisals, resolving any discrepancies through discussion.

### 2.5. Synthesis

A thematic analysis was conducted as a qualitative synthesis to identify key themes, following the three stages described by Thomas and Harden [28]. First, a free line-by-line coding of the findings from primary studies was conducted. Second, new codes were created to organize these “free codes” into related areas, constructing “descriptive” themes. Lastly, the findings of each study were combined into a whole by listing themes and generating analytical themes that described the roles of nurses in achieving high reliability in healthcare within oncology organizations.

## 3. Results

**Figure 1:**
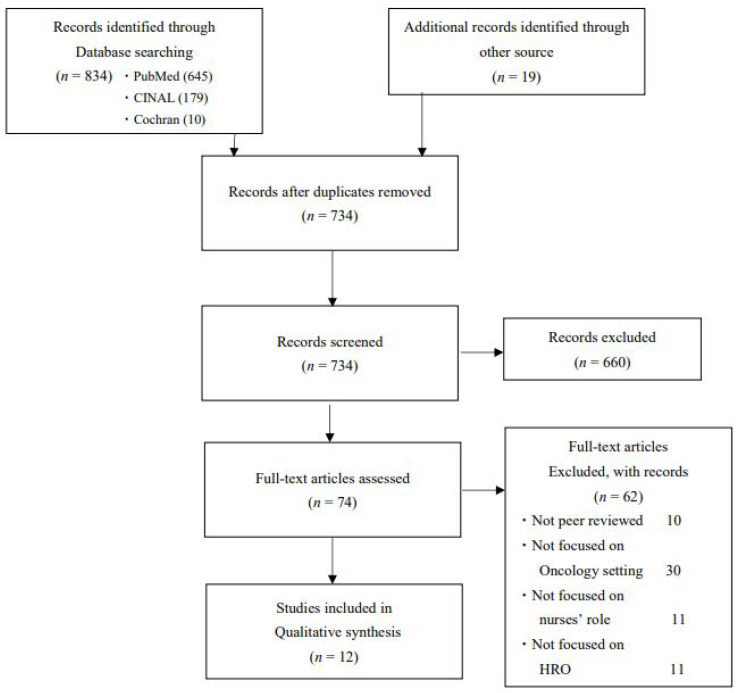
PRISMA flow diagram.

We used the PRISMA 2020 Statement flowchart in the retrieval and selection process, identifying 834 records from an initial database search and an additional 19 records through manual searches of study bibliographies. A total of 734 non-duplicate articles were screened by title and abstract using the standard integrative review process. Following the title and abstract screening, 74 articles were included. These articles underwent full-text review, and 12 were deemed suitable for inclusion (Figure 1).

### 3.1. Characteristics of Included Studies

The characteristics of the 12 included studies are presented in Table 1. The studies were published between 2014 and 2023 and were conducted in the United States (*n* = 9) [11,17,23,24,29,30,31,32,33], Australia (*n* = 1) [34], and Switzerland (*n* = 1) [35], along with a collaborative study spanning four countries (Estonia, Germany, the Netherlands, and the United Kingdom, *n* = 1) [36]. The study approaches primarily included quality improvement projects [17,23,24,29,30,31,32,33], a quantitative quasi-experimental study [11], a quantitative descriptive cross-sectional study [36], and a qualitative study [34].

### 3.2. Quality Assessment

The eight quality improvement studies were assessed using the 16 QI-MQCS criteria. These studies met more than 80% of the quality criteria, with the exception of one study that met 69%, and these studies were therefore rated as having high quality. The remaining four studies were appraised using the MMAT criteria, and all met 100% of the quality standards (Table 1, Supplementary Files S3 and S4).

### 3.3. Themes of Included Studies

Eight themes emerged from the thematic synthesis, representing the crucial role that nurses play in improving organization-wide patient safety and quality care within the field of cancer care: establishing standardized and safe administration; enhancing situational awareness; promoting effective communication; advocating for patients; building a culture of safety; leadership in improving safety culture; staff engagement; patient engagement (Table 2).

### 3.4. Establishing of Standardized and Safe Administration

This theme highlights the establishment of standardized and safe management practices as a fundamental aspect of enabling oncology nurses to provide safe care for patients and their families. Nurses systematically managed the interdependence and risks of cancer treatment safety through strategies involving the enforcement of regulations and multiple safety checks.

#### 3.4.1. Enforcement of Rules and Multiple Safety Checks

To operate the safety management system effectively within their organizations, oncology nurses established universal measures for ensuring compliance with safety protocols and addressing challenges by enforcing rules and implementing multiple safety checks [23,34]. They developed standardized methods for managing the accurate start and end times of chemotherapy within electronic documentation systems and regularly audited these records [23,33]. When setting indicators, the application of nursing-sensitive indicators was particularly effective [24].

#### 3.4.2. Evidence-Based

Cancer treatment often involves combination therapy, which carries a high risk of life-threatening adverse events. As a result, strict management of dosage, administration, and adverse event monitoring is required. Nurses were tasked with regularly measuring, analyzing, and managing safety events using evidence-based methods [23]. This included graphing chemotherapy start and stop times on electronic documentation systems [23], conducting regular causal analyses using statistical process control charts to evaluate incident rates, trends, potential severity, and treatment pathway positions [11]. Rapid, evidence-based discussions were critical for ensuring robust safety measures [30,32].

#### 3.4.3. Promoting the Quality Improvement Process

Nurses enhanced their critical thinking and observation skills to continuously promote the quality improvement process. They strengthened collaboration both within and outside their departments and improved compliance with audits. They documented the exact start and end times of chemotherapy to provide a clear record of treatment progress and ensure continuous audits [23,33]. Nurses also applied critical thinking to determine appropriate line settings, flushing volumes, and rate adjustments [23] and collaborated with vascular access teams to respect their expertise while maintaining sensitivity to operational standards [32].

### 3.5. Enhancing Situational Awareness

#### 3.5.1. Proactive Identification of Patient Conditions

By proactively assessing patient conditions, nurses evaluated preventable harm events, responded to changes in patients, and reduced uncertainty regarding potential outcomes [29]. Huddles were used to strengthen situational awareness and foster a culture of accountability and collaboration through standardized structures and processes for identifying and planning for high-risk patients [29].

#### 3.5.2. Improved Situational Awareness Through Standardized Structure

This goals was met by actively identifying high-risk patients and improving planning through standardized structures and processes. For instance, nurses used Computerized Provider Order Entry (CPOE) systems to ensure a clear understanding of treatment protocols and regularly checked these protocols for accuracy [34]. They also participated in quality and safety culture education programs to increase awareness of high-risk situations [17]. By adopting patients’ perspectives, nurses maintained vigilance in safety management and remained aware of the quality, limitations, and outcomes of organizational teams and services [34].

#### 3.5.3. Increasing Empowerment to Proactively Escalate Their Concerns

To maintain and enhance situational awareness, nurses used huddles to foster accountability and promote proactive problem identification [29]. During these huddles, group meetings were held to identify risk factors affecting individual patients and review patient-specific safety management strategies [32]. These opportunities improved information sharing, empowerment, collaboration, and communication among team members.

### 3.6. Promoting Effective Communication

#### 3.6.1. Openness in Communication

In cancer care settings, promoting effective and structured communication was critical for coordinating relationships among professionals and implementing patient-centered safety measures. By fostering openness in communication, nurses were able to confidently speak up and report dangerous incidents without fear of negative consequences [36]. This openness allowed team members to learn from their own and others’ mistakes, creating a more open atmosphere for organizational learning [36].

#### 3.6.2. Accurate and Structured Communication

Systematic communication tools were used to ensure accurate and structured communication among healthcare professionals [11]. Emphasis was placed on two-way communication, including providing feedback on safety reports and improving the clarity of follow-ups for staff who reported issues [30]. Nurses also developed mechanisms to communicate solutions and successes, ensuring that all healthcare professionals could benefit from the positive outcomes of safety event reviews and promoting the sustainability of improvements [24].

### 3.7. Advocating Patient

#### 3.7.1. Express a Large Variety of Barriers to Speak up Concerns

As patient advocates, nurses took responsibility for ensuring patient safety and acting in patients’ best interests. However, they faced multiple barriers to voicing concerns, such as oppressive hierarchies, fear of job loss, disciplinary actions, harassment, or retaliation [35]. Despite these obstacles, nurses persisted in expressing concerns to protect patients from adverse incidents, even in conditions that encouraged silence [35].

#### 3.7.2. Trade-Off

Nurses navigated role conflicts by engaging in role negotiation during interactions, minimizing the cost of conflict. To reduce barriers to speaking up, they conducted risk assessments and made deliberate trade-offs [35]. For instance, nurses weighed the potential harm of specific patient behaviors against the perceived risks of voicing concerns. This internal calculation involved evaluating the likelihood and severity [35] of harm, often described using terms such as “trade-offs”, “balancing”, “risk-benefit evaluation”, and “weighting” [35].

#### 3.7.3. Establish a Shared Mental Relationship

Nurses developed shared mental models with families to recognize and address risk factors collaboratively [32]. For example, discussions with parents included proper handling and access of central lines, hand hygiene, and standard care elements expected during a child’s hospitalization [32]. By fostering shared mental models, families were better equipped to advocate for their children when care did not meet expectations [32].

### 3.8. Building a Safety Culture

#### 3.8.1. Make a Priority for Safety

To advance safety management, it is essential to transform the nursing culture within institutions. Nursing leadership prioritized the safety of chemotherapy, leading frontline staff to recognize the importance of process changes for their own safety [23]. A clear project champion held nurses accountable for implementing new processes [23], emphasizing the critical importance of accurately determining chemotherapy start and end times [23].

#### 3.8.2. Trust

Fostering trust among staff is crucial to establishing a culture of safety. This involves cultivating the belief that everyone works collaboratively and that safety management focuses on addressing weak processes, not targeting individuals. By enhancing leadership practices, celebrating safety successes, and boosting morale, employees develop trust in the organization’s fair approach to safety events [30]. Corrective actions are perceived as process improvements, while rare disciplinary actions are understood to address deliberate policy violations, not human errors [30].

#### 3.8.3. Establish and Reinforce a “Culture of Voice”

Nurses implemented multifaceted strategies to promote collaboration within their departments [33]. As team members, nurses had the opportunity to learn from both their own and others’ mistakes, cultivating a more open atmosphere for organizational learning [36]. They were encouraged to speak up about issues that could negatively impact the organization without fear of retaliation [11]. Practices like huddles provided opportunities for team members to share concerns, insights, observations, and recommendations for risk mitigation, strengthening a “speak-up” culture [11,32,33].

#### 3.8.4. Promote Collective Commitment

Fostering a collective commitment to safety management is vital [33]. Nurses participated in pre-treatment peer reviews to express concerns and provide feedback, promoting [33] team alignment. By recognizing risk factors, learning from one another, and engaging in discussions with pediatric patients’ parents, nurses encouraged open communication and genuine engagement in safety initiatives [32].

### 3.9. Leadership in Improving a Culture of Safety

#### 3.9.1. Maintain the Direction of the Change

Oncology nurses play a pivotal role in improving safety culture by setting a clear direction for change, aligning staff, and inspiring motivation [30]. They conducted safety meetings and walk-arounds to share direct experiences of their unit’s strengths, vulnerabilities, and errors, incorporating safety reporting into daily practice [30]. By addressing team dynamics, interpersonal relationships, and system-level issues, nurses supported the operation of safety committees and provided accessible self-reporting mechanisms [30]. Timely feedback, including real-time data on processes and outcomes, helped to identify areas needing improvement while promoting sustainability through engagement [24].

#### 3.9.2. Create Education and Encourage for Staff

Nurse leaders organized regular education programs to instill a culture of safety among staff. They developed tools to share safety management processes and findings with frontline staff and set annual safety management goals in collaboration with team members [24]. By fostering adherence to safety regulations, leaders supported a safer, higher-quality healthcare delivery system [31]. Feedback and encouragement further sustained this safety culture [35].

#### 3.9.3. Audit and Maintain a Culture of Safety

Nurse leaders conducted regular safety rounds to ensure effective safety management [32]. These rounds included harm prevention huddles, where leaders collaborated with caregivers, physicians, frontline staff, and patients to align accountability and prevention strategies [32]. Leadership maintained a visible presence and consistently refocused discussions on sustaining safety processes [32].

#### 3.9.4. Servant Leadership

Nurse leaders demonstrated inclusivity by valuing the contributions of others [35]. As servant leaders, they listened empathetically to staff, focusing on process improvements and seeking solutions to barriers [31]. Leaders reassured staff that individual shortcomings would not be targeted and prioritized the implementation of viable ideas proposed by frontline professionals [31]. This approach encouraged collective commitment to safety and collaboration among team members [31].

### 3.10. Staff Engagement

#### 3.10.1. Education and Coaching

Nurses implemented education modules and training programs to provide effective learning opportunities for frontline staff [31]. They used findings from safety unit surveys to share unit-specific progress and facilitate discussions about barriers and solutions with staff. Rounds utilizing “K-cards” promoted staff and patient engagement, clarifying roles and expectations for improvement initiatives [31].

#### 3.10.2. Encourage Staff to Be Involved

Nurses fostered inclusivity by encouraging staff participation, even for those unable to formally join committees [30]. This approach broadened engagement while reinforcing equal responsibility for safety matters. By maintaining consistent support and creating a respectful environment, nurses cultivated an open reporting culture with continuous staff involvement [30]. Unit-specific survey findings were shared to facilitate discussions and promote quality improvement [31].

### 3.11. Patient Engagement

#### Promoting Communication Between Patients and the Team

Nurses facilitated appropriate communication between patients and healthcare teams. During patient rounds and consultations, feedback was gathered and discussed with leadership teams and at safety meetings [24,30]. This process aimed to enhance patients’ and families’ self-efficacy by involving them in safety management efforts and leveraging their knowledge and skills in self-care.

## 4. Discussion

This review demonstrates that nurses play a central role in identifying, communicating, and correcting safety threats when establishing highly reliable organizations in cancer care facilities. In addition, nurses play a leading role in patient advocacy and collective agency for patient safety, as well as in encouraging healthcare staff, patients, and families to establish a culture of safety in an organization.

Although the protocol-based management of anticancer drug treatment through an electronic medical record system reduces ordering errors [37], changes in medication schedules and volumes can still result in errors if the administration is not appropriately adjusted to reflect these changes [38]. In addition, anticancer treatment has identified two inter-related overarching types of interdependencies associated with chemotherapy: those related to the organization of clinical activities and those inherent to chemotherapy regimens, which dictate precise combinations of medications, as well as the integration of medications with specific tests [34]. Weingart et al. [38] conducted a review of chemotherapy errors and reported that prospective risk assessments are effective tools for understanding and improving oral and infusion chemotherapy processes. In this review, nurses play a crucial role in ensuring the safety of chemotherapy by performing multiple safety checks to manage its complex processes. Tools like the PDCA cycle and audits are effective for identifying risks and improving safety in both oral and infusion chemotherapy.

By proactively assessing the patient’s condition, nurses assessed preventable patient harm events, responded to changes in the patient, and reduced the uncertainty of possible outcomes. Nurses were responsible for verifying each dose against the protocol to notify physicians of any discrepancies [29]. Huddles were used to strengthen situational awareness and foster a culture of accountability and collaboration through standardized structures and processes to proactively identify and plan for high-risk patients.

To reduce patient safety risks in cancer treatment, which is characterized by interdependence, nurses enhanced their proactive situational awareness of patients’ conditions. Situational awareness—“knowing what’s going on” [39]—is fundamental to the dynamic reasoning and decision-making processes [40] required by healthcare providers in response to evolving clinical situations. Nurses combined and interpreted multiple sources of information to determine the meaning and relevance of clues and information about patient safety risks, and they proactively predicted near-term situational events and dynamics. For example, huddle meetings were held within a department to share information to identify patient risks early and share a systematic process for escalating safety care [29].

Communication breakdown among healthcare professionals has been identified as a major threat to patient safety and errors [41]. In oncology, a multifaceted and highly collaborative field of healthcare in which even minor errors can lead to severe patient harm, effective and assertive communication among team members about errors and risky behaviors is crucial [35].

Talking about patient safety is vital to avoid errors reaching the patient, thus preventing harm and improving the culture of teamwork and safety [35]. However, in clinical settings, there are several barriers to speaking up about patient safety [35,42,43]. Thus, nurses should use systematic communication tools to promote accurate and structured communication between healthcare professionals. Careful examination of the complex factors that enable or hinder nurses’ ability to take assertive action for patient safety is essential for improving interdisciplinary communication and developing and sustaining safe patient care systems [44].

Nurses play the critical role of “patient advocates”, acting in the best interest of the patient. However, multiple barriers to nurses’ ability to assert their concerns persist in healthcare settings, including oppressive hierarchies and fears of job loss, discipline, harassment, or retribution [44,45]. These barriers often lead to significant role conflict for nurses, as their competing role expectations may demand opposing behaviors such as prioritizing self-protection over advocating patient safety [44,46].

To overcome these challenges, nurses made concerted efforts to voice their concerns, highlighting the barriers to speaking up and the conditions that foster a culture of “silence”, all while striving to protect patients from harm [35]. Despite these obstacles, nurses persisted in their advocacy efforts not only by speaking out but also by helping families advocate for patients when care fell short of expectations. Schwappach et al. [35] conceptualized the influence of both motivations and barriers on the decision-making process regarding speaking up. Nurses weigh the trade-offs of speaking up or remaining silent by considering factors such as “judging the level of risk”, “differing perceptions of harm between professions”, “anticipation of negative outcomes”, and “predictability of the actor’s response”. The process of evaluating concerns about risks is complex and requires a relative interpretation of safety rules. The relative interpretation of safety rules created dissonance among some oncology nurses, leading to feelings of resignation and futility, often referred to as “acquiescent silence” in the organizational silence literature. Therefore, to encourage nurses’ decisions to speak up, nurse leaders must take the initiative to voice safety concerns themselves and work to eliminate the sense of powerlessness among nursing staff associated with “acquiescent silence”. Furthermore, by fostering a shared understanding and partnership among patients, their families, and healthcare providers, nurses strengthen the foundation of patient advocacy. To achieve this, nurses must provide patients and their families with clear and specific guidance on appropriate self-care, while also building trusting relationships to ensure that patients feel comfortable expressing their concerns and anxieties. Additionally, by offering anticipatory guidance on potential risks, nurses can enhance patients’ ability to respond effectively to unforeseen situations [32].

Nurses were not only responsible for ensuring patient safety on an individual level but also responsible for fostering a culture of open communication within their organizations. This involved consistently raising concerns and asking questions of organizational management, supporting one another as team members, and cultivating a shared culture that rejected criticism or undue pressure from superiors or colleagues. A key component of patient safety is an organizational culture in which all clinicians have individual and collective authority to question the plan of care and power to “stop the line” [47] or change the direction of a clinical situation in the patient’s best interest [44]. With chemotherapy safety prioritized by the nursing leadership, frontline staff accepted that this process change was important for their safety. Strategies for implementing these integrated safety approaches included identifying downwards drift in practice standards, or “normalization of deviance”, as part of the organizational culture [44].

Building a culture of safety requires a foundation of trust among staff. This meant fostering a collaborative environment in which everyone worked together and approached safety management as a process of identifying weak systems rather than viewing staff as weak individuals prone to failure. Trust is the cornerstone of strong safety culture. Additionally, nurses were given opportunities to learn from both their own mistakes and those of others, cultivating an organizational learning atmosphere. This openness encouraged all the “crew members” to share their concerns, insights, positive observations, and recommendations for improvement. These practices reinforced a “culture of voice”, promoting proactive communication and continuous enhancement of safety practices.

High reliability requires systems that consistently create conditions for error-free work supported by well-defined policies and standard procedures. However, these systems are only effective when paired with a specific organizational culture [48,49]. This safety culture is characterized by a sense of trust, accountability, and psychological safety [49,50,51]. The review found that nurses aim to drive cultural changes across an organization for multimodal high-reliability organization initiatives. The multimodal implementation plan for achieving high reliability encompasses (1) safety science and error prevention training, (2) safety coaching, (3) proactive safety rounds, (4) quality improvement and analytics training, (5) local learning initiatives, and (6) a just culture algorithm grounded in appreciative inquiry to strengthen the root cause analysis process [49]. For instance, nurses not only delivered safety coaching and conducted regular safety rounds but also served as key advocates for safety projects, fostering trust and accountability within the team. Nurses proactively share committee findings and decisions during weekly team training huddles, providing an opportunity for the entire team to voice additional concerns, offer insights, give positive recognition, and suggest mitigation strategies [11]. They work to strengthen and sustain a culture of safety by fostering trust among team members, enhancing psychological safety, and promoting a “culture of voice”, in which open communication and proactive engagement are encouraged and valued.

Promoting the five principles of high-reliability organizations requires cultivating a robust safety culture across the organization. Since leaders are the primary drivers of organizational culture [52], it is crucial for executives to demonstrate a strong commitment to establishing both a safety culture and high-reliability organization principles. Staff, including frontline members, should actively engage in situational awareness, share critical information, and respect their expertise regardless of their rank. This approach reflects “inclusive leadership”, where the diverse perspectives and actions of all members contribute to the creation of an HRO. It also aligns with “servant leadership”, wherein top management delegates aspects of leadership to achieve shared goals effectively. The findings of this review revealed that these leadership elements are particularly evident in the context of cancer nursing, where fostering collaboration and mutual respect are essential for maintaining high standards of safety and care.

Professional development and engagement in healthcare are essential for strengthening the foundations of high-reliability organizations and driving complex organizational transformation [53]. Leaders can guide their organizations toward a safer future by setting a clear goal of zero harm and fostering an environment that prioritizes teamwork, communication, learning, and continuous improvement [53]. In this review, nurses had the opportunity to reflect on failures, analyze lessons learned, and discuss barriers and potential solutions, thereby raising their awareness of patient safety by promoting equal participation and shared responsibility in meetings, ensuring consistent support among team members, and maintaining a respectful and inclusive environment. In other words, nurses demonstrated inclusive leadership by practicing empathetic listening and encouraging staff to identify barriers and propose solutions, emphasizing process improvement rather than assigning blame to individuals. Additionally, nurses exhibited servant leadership by embracing the concerns and ideas raised by frontline nurses, fostering connections within the team, and discussing what collective actions could be taken to reduce harm. Together, they worked as a united team to promote safety initiatives. In the field of oncology, interdisciplinary teams are formed under the treatment plans established by oncologists to enhance therapeutic outcomes. However, when a strong paternalistic approach by oncologists is present, the risks, concerns, and opinions of frontline staff may remain unvoiced during daily operations due to fears of alienation or criticism. Nurse leaders must continue to value the unique qualities of nursing staff and foster a culture that respects and encourages their opinions on safety risks.

Furthermore, nurses were reminded of the importance of continued involvement in patient safety by ensuring that everyone had an equal voice and equal responsibility in meetings, consistent support from team members, and the experience of maintaining a respectful environment. Psychological safety is a critical factor in fostering staff engagement among team members, including physicians [54]. The benefits include improved employee well-being, enhanced job satisfaction, reduced workplace stress, and increased interest in and commitment to quality improvement initiatives. By creating an environment that values learning from mistakes, psychological safety drives both personal and organizational growth [54].

Patient engagement is critical for safety. Involving patients and families in care can prevent adverse events and the inappropriate use of medication [55,56]. The world has emphasized the importance of patient engagement and safety [57,58]. Nurses play a pivotal role in ensuring patient safety [59]. Nurses play a pivotal role in facilitating patient participation in the development of high-reliability organizations for patient safety. Nurses should use patient participation as a learning process, assist patients in their own care, and avoid adopting authoritarian approaches that may discourage participation [60,61]. Nurses engaged and educated patients and family members to raise awareness about the safety of their own care to guide the establishment and improvement of patient participation in patient safety.

### Strengths and Limitations

This review had several limitations. First, we conducted a literature search across three well-known databases; however, we cannot definitively conclude that all studies related to HRO and cancer nursing were identified and included. It may be necessary to expand the search to additional databases and unpublished materials. Second, due to the limited number of eligible studies, we had to incorporate different types of studies to synthesize evidence, which also required applying various methods to assess the quality of each study. Third, 9 of the 12 studies included in the review were conducted in the United States, and the remaining three were conducted in Europe and Australia. This compromises the generalizability of the findings as the number of countries captured is insufficient for the entire world. Fourth, this study included only papers published in English, which may have excluded relevant evidence published in other languages. Fifth, this review included studies conducted in populations with diverse cancer types and in diverse cancer care settings. Further research is needed to determine the characteristics of the target cancer populations and cancer care settings. In pediatric oncology settings, it is particularly important to focus on risks affecting both children and their families. Additionally, since families play a critical role in supporting ongoing treatment and self-care, their engagement in safety measures is essential. Therefore, further research is needed to address these unique characteristics of the treatment environment. Finally, the role of nurses may vary depending on their expertise, such as oncology, geriatric, and advanced practice nurses. Therefore, promoting research that considers these subspecialties is necessary. Advanced practice nurses viewed themselves as role models and leaders for other healthcare staff, leveraging their expertise and professional experience to gain a broader perspective on healthcare [62]. Therefore, future research should focus on advanced practice nurses (APNs) being identified as change agents at the system level due to their decision-making abilities and their connectivity within multi-professional teams.

Although this review has some limitations, we believe it has paved the way for further consideration of the important but under-represented issue of the role of nurses in high reliability in oncology care.

## 5. Conclusions

With the increasing number of high-risk patients undergoing interventions, ensuring high-quality care in a safe environment has become increasingly challenging, highlighting the need to establish highly reliable organizations. To address this, we conducted a systematic review and thematic analysis to identify and examine the critical role of nurses in promoting high reliability within oncology organizations, with the aim of enhancing patient safety and outcomes.

Nurses play a crucial role in identifying, communicating, and addressing safety threats; overcoming barriers to safety within their organizations; and fostering a culture of safety alongside patients and families. Patient safety culture, as reported by cancer nurses, varies across European countries, with contextual factors such as recognition of the nursing role and education significantly influencing it [36].

Moreover, the role of nurses in promoting safety is expected to vary according to their years of experience and professional position, such as staff nurses, advanced practice nurses, and nursing administrators. While this review analyzed a relatively small sample of the literature, the findings highlight the need for further research that considers the unique characteristics of health systems and healthcare delivery contexts.

## Figures and Tables

**Table 1:** The characteristics of the 12 included studies.

Author(s) and Year of Publication	Study Population and Setting	Objective(s)	Design	Methods	Themes of Nurses’ Role *	QI-MQCS Score,%	MMAT Score,%
Evans et al. (2021) [29]	The Medical Emergency Team involved nurses; physicians, including residents, hospitalists, and Pediatric Intensive Care Unit representatives; and the pediatric inpatient acute care setting (i.e., medical–surgical, hematology-oncology, and intermediate care units), Arkansas Children’s Hospital, USA. Number of nurses; not noted.	To describe implementation of a program to improve recognition and response to clinical deterioration within the pediatric inpatient acute care setting.	Implementation study quality improvement project	Assembled an inter-professional team to evaluate preventable patient harm events. Evaluated an existing situational awareness framework incorporating principles of high-reliability organizations and subsequently implemented a program reducing emergency transfers.	B	94	-
Lichtner et al. (2020) [34]	Healthcare professionals, doctors (n = 10), nurses (n = 6), a pharmacist, and oncology CPOE team members (n = 2) in the oncology unit of a 350-bed tertiary pediatric hospital in New South Wales, Australia.	To investigate how computerized provider order entry (CPOE) for chemotherapy relates to other safety strategies in a pediatric clinical oncology unit, with a focus on the management of interdependencies.	Multi-method qualitative study	Analyzed 827 oncology incidents reported following CPOE implementation and carried out semi-structured interviews. Results were interpreted according to safety models.	A, B	-	100
Looper et al. (2016) [23]	A multidisciplinary task force: physicians, advanced practice nurses, nursing leadership, staff nurses, pharmacy, nurse educators, and clinical research associates, St. Louis Children’s Hospital, USA Number of nurses; not noted.	To develop best practices for multidisciplinary teams managing chemotherapy for children and evaluate the process of their implementation.	Implementation study quality improvement project	Established best practices for nurses who administer chemotherapy to children. Focused on the establishment of standardized and safe administration techniques, exact administration times, and consistent electronic documentation.	A, E	81	-
Plouff et al. (2022) [30]	The leadership team included physicians, advanced practice providers, nurses, and business and administrative leaders at MD Anderson Cancer Center in Sugar Land, Houston, USA Number of nurses; not noted.	To review the Secure, Attentive, Focused, Engaged (SAFE^TM^) initiative to create, foster, and continuously improve safety culture and its impact on our safety culture and patient experience.	Implementation study quality improvement project	The SAFE^TM^ initiative was conceptualized and implemented by the leadership team. Recorded quantitative measures and qualitative improvements, such as increased engagement and improved staff morale.	A, C, E, F, G, H	88	-
Salinas et al. (2022) [24]	Hospital staff (nurses, nurse manager and clinical nurse leader) within the surgical cohort, Thoracic and Cardiovascular Surgery, The University of Texas MD Anderson Cancer Center, Houston, USA. Number of nurses; not noted.	To evaluate the effect of K-Card rounding on key quality measures such as patient experience and NSI, as well as to standardize workflows related to leadership rounding and process auditing.	Implementation study quality improvement project	Developed nurse sensitivity indicator prevention bundles, root cause analysis tools, and best practices. Implemented K-Cards to standardize processes, engage patients in their care, and promote staff identification of barriers and solutions. Reviewed data in the unit leadership operations committee meeting for the effectiveness of K-Card Interventions.	A, C, F, H	81	-
Salinas et al. (2022) [31]	Hospital staff (nurses, nurse manager, clinical nurse leader and associate directors) within the surgical cohort, Thoracic and Cardiovascular Surgery, The University of Texas MD Anderson Cancer Center, Houston, USA. Number of nurses; not noted.	To increase the percentile rank for responsiveness of hospital staff within the surgical cohort to 80%.	Implementation study quality improvement project	Developed Responsiveness K-cards. Analyzed process barriers using observation, active listening, and interpretive inquiry. Descriptive analysis of percentile rank for responsiveness of staff.	F, G	88	-
Schwappach et al. (2014) [35]	Doctors (senior doctor, 4, resident, 10) and nurses (head nurse, 3, nurse, 15) at the ambulatory oncology units or on the wards, who had sufficient working experience in oncology, of six hospitals with seven oncology departments, Switzerland.	To explore factors that affect oncology staff’s decision to voice safety concerns or to remain silent and to describe the trade-offs they make.	Qualitative interview study	Semi-structured interviews with experienced oncology staff. Questions about how often participants experience situations that they felt would require voicing their concern and whether they generally feel comfortable to raise patient safety issues with co-workers and supervisors in their team.	D, F	-	100
Sharp et al. (2019) [36]	Eligible participants were 393 cancer nurses from the four countries (Estonia, Germany, the Netherlands, and the United Kingdom) involved. Data collection was conducted anonymously on a voluntary basis during annual conferences of the National Cancer Nursing Societies in each country.	To explore the differences in perceived patient safety cultures among cancer nurses working in four European countries.	An exploratory cross-sectional study	Investigated workplace patient safety culture among cancer nurses in four European countries using the Hospital Survey on Patient Safety Culture (HSPSC).	C, E	-	100
Swanson et al. (2021) [11]	The department in this study was one of five national oncology hospital-based radiation oncology (RO) departments. The department staff comprised radiation oncology physicians, medical physicists, radiation therapists, nurses, and support personnel and were based in the USA. Number of nurses; not noted.	To describe the development, implementation, and impact of a six-month, two-pronged team training and incident-learning intervention adapted from crew resource management (CRM) principles on the rate of incident reporting in a complex radiation oncology setting.	Quasi-experimental study	The development and implementation of a twostep custom CRM training and incident learning system (ILS) intervention. Data collected over the six-month study period included the monthly number and rate of incidents reported, patient treatments per workload, incident severity potential, and the step or location of incident origination.	A, C, E	-	100
Vijayakumar S et.al. (2019) [33]	A multidisciplinary group’s consensus peer-review members included physicians, therapists, physicists, dosimetrists, and nurses working at the Radiation Oncology Department, University of MS Medical Center, Jackson, MS, USA. Number of nurses; not noted.	To achieve the goal of “chase zero”, the authors conducted a pre-treatment and multidisciplinary group consensus peer-review (GCPR) program in radiation oncology to identify its effectiveness.	Implementation study quality improvement project	Developed the goal of “chasing zero harm” and implemented the GCPR model program. Analyzed for department approval target coverage and dose constraints.	E	69	-
Willis et al. (2023) [32]	A multidisciplinary team of physicians, nurse practitioners, nursing and physician leadership, frontline staff, and improvement specialists in a large free-standing tertiary care children’s hospital, St. Louis, USA. Number of nurses; not noted.	To describe the quality improvement initiative to eliminate Central Line-Associated Bloodstream Infections (CLABSIs) in the pediatric oncology population at our institution.	Implementation study quality improvement project.	Created a multidisciplinary team, being mindful to identify roles and responsibilities upfront. Developed a key driver diagram and designed and implemented interventions to influence our primary outcome. Implemented interventions and conducted Plan–Do–Study–Act cycles concurrently.	A, B, D, E, F	88	-
Woodhouse et al. (2016) [17]	The Department of Radiation Oncology at the University of Pennsylvania, USA, including approximately 39 physicians, 43 medical physicists, 30 dosimetrists, 28 nurses, and 82 radiation therapists,	To describe advancing a large, multisite radiation oncology department toward high reliability through the implementation of a comprehensive safety culture (SC) program.	Implementation study quality improvement project	Implemented multifaceted safety initiatives at the main academic center and across all network sites. Analyzed a comparison of pre- and post-intervention data using a control chart.	B	94	-

* Themes of nurses’ role: A. establishing standardized and safe administration; B. enhancing situational awareness; C. promoting effective communication; D. advocacy of patient; E. building safety culture; F. leadership in improving culture of safety; G. staff engagement; H. patient engagement.

**Table 2:** Themes and sub-themes representing nurses’ roles.

Theme	Subtheme
A. Establishing standardized and safe administration	A-1. Enforcement of rules and multiple safety checks [23,24,34]
	A-2. Evidence-based [11,23,30,32]
	A-3. Promoting the quality improvement process [23,24,32]
B. Enhancing situational awareness	B-1. Proactive identification of patient conditions [29]
	B-2. Improved situational awareness through standardized structure [17,34]
	B-3. Increasing empowerment to proactively escalate their concerns [29,32]
C. Promoting effective communication	C-1. Openness in communication [36]
	C-2. Accurate and structured communication [11,24,30]
D. Advocacy of patient	D-1. Expresses a large variety of barriers to address concerns [35]
	D-2. Trade-off [35]
	D-3. Establish a shared mental relationship [32]
E. Building safety culture	E-1. Make a priority for safety [23]
	E-2. Trust [30]
	E-3. Establish and reinforce a “culture of voice” [11,32,33,36]
	E-4. Promote collective commitment [32,33]
F. Leadership in improving culture of safety	F-1. Maintain the direction of the change [24,30]
	F-2. Create education and encourage for staff [24,31,35]
	F-3. Audit and maintain a culture of safety [32]
	F-4. Servant leadership [31,35]
G. Staff engagement	G-1. Education and coaching [31]
	G-2. Encourage staff to be involved [30,31]
H. Patient engagement	H-1. Promoting communication between patients and the team [24,30]

## Data Availability

The datasets used or analyzed in the current study are available from the corresponding author upon reasonable request.

## Supplementary Materials

The following supporting information can be downloaded at https://www.mdpi.com/article/10.3390/healthcare13030283/s1, Supplementary File S1. Keywords; Supplementary File S2. Searches; Supplementary File S3. Scores; Supplementary File S4. Scores. The systematic review protocol was registered in UMIN Clinical Trials Registry (UMIN000056140). https://center6.umin.ac.jp/cgi-open-bin/ctr/ctr_view.cgi?recptno=R000064146 (accessed on 18 January 2025).
