# Supplementary material for: Enhancing High Reliability in Oncology Care: The Critical Role of Nurses—A Systematic Review and Thematic Analysis
Source: Healthcare (Basel). 2025 Jan 31;13(3):283. doi: 10.3390/healthcare13030283 (PMC11817837; doi:10.3390/healthcare13030283)
Supplement: Supplementary file 1 [file healthcare-13-00283-s001.zip › supplimental.file4.250112.docx]

| Spplementary Flie S4. Scores | | | | | | | | | |
| --- | --- | --- | --- | --- | --- | --- | --- | --- | --- |
| Reference | S1. Are there clear research questions? | S2. Do the collected data allow to address the research questions? | 1.Qualitative | 1.1 Is the qualitative approach  appropriate to answer the question? | 1.2 Are the qualitative data collection methods adequate to the address the research question? | 1.3. Are the findings adequately derived from the data? | 1.4. Is the interpretation of results sufficiently substantiated by data? | 1.5. Is there coherence between qualitative data sources, collection,  analysis and interpretation? |  |
|  |  |  | 2. Quantitative randomized controlled trials | 2.1. Is randomization appropriately performed? | 2.2. Are the groups comparable at baseline? | 2.3. Are there complete outcome data? | 2.4. Are outcome assessors blinded to the intervention provided? | 2.5. Did the participants adhere to the assigned intervention? |  |
|  |  |  | 3.Quantitative non-randomized | 3.1. Are the participants representative of the target population? | 3.2. Are the measurements appropriate regarding both the outcome and intervention (or exposure)? | 3.3. Are there complete outcome data? | 3.4. Are the confounders accounted for in the design and analysis? | 3.5. During the study period is the intervention administered (or exposure occurred) as intended? |  |
|  |  |  | 4.Quantitative descriptive | 4.1. Is the sampling strategy relevant to address the research question? | 4.2. Is the sample representative of the target population? | 4.3. Are the measurements appropriate? | 4.4. Is the risk of nonresponse boas low? | 4.5. Is the statistical analysis appropriate to answer the research question? |  |
|  |  |  | 5. Mixed methods | 5.1. Is there an adequate rationale for using a mixed methods design to address the research question? | 5.2. Are the different components of the study effectively integrated to answer the research question? | 5.3. Are the outputs of the integration of qualitative and quantitative components adequately interpreted? | 5.4. Are divergences and inconsistencies between quantitative and qualitative results adequately addressed? | 5.5. Do the different components of the study adhere to the quality criteria of each tradition of the methods involved? |  |
| Lichtner et al.,2020 | YES | YES | 1 | YES | YES | YES | YES | YES |  |
| Schwappach al.,2019 | YES | YES | 1 | YES | YES | YES | YES | YES |  |
| Sharp et al.,2019 | YES | YES | 4 | YES | YES | YES | YES | YES |  |
| Swanson et al.,2021 | YES | YES | 3 | YES | YES | YES | YES | YES |  |
