# Supplementary material for: Enhancing High Reliability in Oncology Care: The Critical Role of Nurses—A Systematic Review and Thematic Analysis
Source: Healthcare (Basel). 2025 Jan 31;13(3):283. doi: 10.3390/healthcare13030283 (PMC11817837; doi:10.3390/healthcare13030283)
Supplement: Supplementary file 1 [file healthcare-13-00283-s001.zip › supplementary.file3.250112.docx]

|  | **1. Organisational motivation** | **2. Intervention rationale** | **3. Intervention description** | **4. Organisational characteristics** | **5. Implementation** | **6. Study design** | **7. Comparator** | **8. Data source** | **9. Timing** | **10. Adherence /fidelity** | **11. Health outcomes** | **12. Organisational readiness** | **13. Penetration/reach** | **14. Sustainability** | **15. Spread** | **16. Limitations** | **Total scores, (%)** |
| --- | --- | --- | --- | --- | --- | --- | --- | --- | --- | --- | --- | --- | --- | --- | --- | --- | --- |
| **Evans et al. (2021)** | + | + | + | + | + | + | + | + | + | + | + | + | - | + | + | + | 15 (94) |
| **Looper et al. (2016)** | + | + | + | + | + | + | + | + | + | + | - | + | - | + | + | - | 13 (81) |
| **Plouff et al. (2022)** | + | + | + | + | + | ＋ | + | + | + | + | - | + | - | + | + | + | 14 (88) |
| **Salinaset al. (2022a)** | + | + | + | - | + | + | + | + | + | + | + | + | - | - | + | + | 13 (81) |
| **Salinaset al. (2022b)** | + | + | + | - | + | + | ＋ | + | + | + | + | + | + | - | - | + | 14 (88) |
| **Vijayakumar et.al. (2019)** | + | + | + | + | + | + | - | + | + | + | - | + | - | + | - | - | 11 (69) |
| **Willis et al. (2023)** | + | + | + | - | + | + | + | + | + | + | + | + | - | + | + | - | 14 (88) |
| **Woodhouse et al. (2016)** | + | + | + | + | + | + | + | + | + | + | + | + | + | - | + | + | 15 (94) |

+ met - unmet
