# Supplementary material for: Enhancing High Reliability in Oncology Care: The Critical Role of Nurses—A Systematic Review and Thematic Analysis
Source: Healthcare (Basel). 2025 Jan 31;13(3):283. doi: 10.3390/healthcare13030283 (PMC11817837; doi:10.3390/healthcare13030283)
Supplement: Supplementary file 1 [file healthcare-13-00283-s001.zip › supplementary.file2.250112.docx]

**Supplementary file 2. Search Results**

The 283 results were searched for the keywords " High Reliability Organizations " and the keywords of "role of nurse (nurse)" in each database, and the results were removed from duplicates

| **PubMed** | | 2024/2/28 |
| --- | --- | --- |
| Search number | Query | records |
| #1 | "High Reliability Organizations"[MeSH] OR "high reliability organiz*" OR "mindful organiz*" | 298 |
| #2 | "high reliability" OR "HRO" | 6,721 |
| #3 | "Oncology Service, Hospital"[Mesh] OR "Health Facilities"[Mesh] OR "health care" | 1,859,320 |
| #4 | oncology or cancer | 5,201,904 |
| #5 | hospital OR "care unit" OR organization OR facilit* OR institution OR department | 21,722,949 |
| #6 | #4 AND #5 | 3,550,258 |
| #7 | #3 OR #6 | 5,271,127 |
| #8 | #2 AND #7 | 1,188 |
| #9 | #1 OR #8 | 1,292 |
| #10 | Search: "Oncology Nursing"[MeSH] OR "Nurse's Role"[MeSH] OR Nurses OR "Health Personnel" OR "role of nurse" OR "Nursing Role" OR "Professional Role" OR "Medical Personnel" OR "Health Care Provider" OR "Healthcare Worker" OR "Health Care Professional" OR "Health Care Staff" | 657,913 |
| #11 | #9 AND #10 | 206 |

| **CINAHL**  ※"+" includes a sub-word, and if it is not attached, there is no sub-word | | 2024/2/28 |
| --- | --- | --- |
| Search number | Query | records |
| S1 | "high reliability organiz*" OR "mindful organiz*" | 206 |
| S2 | "high reliability" OR "HRO" | 2,085 |
| S3 | ( (MH "Oncology Care Units") OR (MH "Cancer Care Facilities") OR (MH "Health Facilities+") ) OR "health care" | 1,038,749 |
| S4 | oncology OR cancer | 582,149 |
| S5 | hospital OR "care unit" OR organization OR facilit* OR institution OR department | 1,237,947 |
| S6 | S4 AND S5 | 84,932 |
| S7 | S3 OR S6 | 1,096,230 |
| S8 | S2 AND S7 | 513 |
| S9 | S1 OR S8 | 581 |
| S10 | ( (MH "Oncology Nursing+") OR (MH "Nursing Role") OR (MH "Professional Role+") OR (MH "Nurses+") OR (MH "Health Personnel+") ) OR "role of nurse" OR "Nurse's Role" OR "Health Care Staff" OR "Medical Personnel" OR "Health Care Provider" OR "Healthcare Worker" OR "Health Care Professional" | 733,267 |
| S11 | S9 AND S10 | 136 |

| **Cochrane Library** | |  |  |
| --- | --- | --- | --- |
| Search number | Query | records | (Trials) |
| #1 | MeSH descriptor: [High Reliability Organizations] explode all trees | 0 | 0 |
| #2 | (high reliability NEXT organiz*) | 2 | 2 |
| #3 | (mindful NEXT organiz*) | 0 | 0 |
| #4 | #1 OR #2 OR #3 | 2 | 2 |
| #5 | high reliability OR "HRO" | 277 | 269 |
| #6 | MeSH descriptor: [Oncology Service, Hospital] explode all trees | 27 | 27 |
| #7 | MeSH descriptor: [Health Facilities] explode all trees | 23,372 | 23,163 |
| #8 | health care | 90,922 | 88,185 |
| #9 | #6 OR #7 OR #8 | 109,073 | 106,233 |
| #10 | oncology OR cancer | 254,455 | 250,789 |
| #11 | hospital OR "care unit" OR organization OR facilit* OR institution OR department | 717,867 | 704,308 |
| #12 | #10 AND #11 | 88,865 | 85,441 |
| #13 | #9 OR #12 | 191,120 | 185,713 |
| #14 | #5 AND #13 | 31 | 26 |
| #15 | #4 OR #14 | 32 | 27 |
| #16 | MeSH descriptor: [Oncology Nursing] explode all trees | 227 | 226 |
| #17 | MeSH descriptor: [Nurse's Role] explode all trees | 617 | 617 |
| #18 | Nurses OR "Health Personnel" OR "role of nurse" OR "Nursing Role" OR "Professional Role" OR "Medical Personnel" OR "Health Care Provider" OR "Healthcare Worker" OR "Health Care Professional" OR "Health Care Staff" | 28,488 | 26,903 |
| #19 | #16 OR #17 OR #18 | 28,922 | 27,337 |
| #20 | #15 AND #19 | 3 | 1 |
|  |  |  |  |
|  | **136 (CINAHL) + 206(PubMe) +1(Cochrane) exclude duplicates** | **283** |  |

The 451 results were searched for the keywords " High Reliability Organizations " and the keywords of "oncology" in each database, and the results were removed from duplicates

| **PubMed** | | 2024/2/28 |
| --- | --- | --- |
| Search number | Query | records |
| #1 | "High Reliability Organizations"[MeSH] OR "high reliability organiz*" OR "mindful organiz*" | 298 |
| #2 | "high reliability" OR "HRO" | 6,721 |
| #3 | "Oncology Service, Hospital"[Mesh] OR "Health Facilities"[Mesh] OR "health care" | 1,859,320 |
| #4 | oncology or cancer | 5,201,904 |
| #5 | hospital OR "care unit" OR organization OR facilit* OR institution OR department | 21,722,949 |
| #6 | #4 AND #5 | 3,550,258 |
| #7 | #3 OR #6 | 5,271,127 |
| #8 | #2 AND #7 | 1,188 |
| #9 | #1 OR #8 | 1,292 |
| #10 | oncology OR Neoplasms[MeSH] OR cancer OR Tumor OR Malignan* | 5,863,273 |
| #11 | #9 AND #10 | 439 |

| CINAHL　※"+" includes a sub-word, and if it is not attached, there is no sub-word | | 2024/2/28 |
| --- | --- | --- |
| Search number | Query | records |
| S1 | "high reliability organiz*" OR "mindful organiz*" | 206 |
| S2 | "high reliability" OR "HRO" | 2,085 |
| S3 | ( (MH "Oncology Care Units") OR (MH "Cancer Care Facilities") OR (MH "Health Facilities+") ) OR "health care" | 1,038,749 |
| S4 | oncology OR cancer | 582,149 |
| S5 | hospital OR "care unit" OR organization OR facilit* OR institution OR department | 1,237,947 |
| S6 | S4 AND S5 | 84,932 |
| S7 | S3 OR S6 | 1,096,230 |
| S8 | S2 AND S7 | 513 |
| S9 | S1 OR S8 | 581 |
| S10 | (MH "Neoplasms+") OR oncology OR cancer OR Tumor OR Malignan* | 937,109 |
| S11 | S9 AND S10 | 43 |

| **Cochrane Library** | |  | (Trials) |
| --- | --- | --- | --- |
| Search number | Query | records | records |
| #1 | MeSH descriptor: [High Reliability Organizations] explode all trees | 0 | 0 |
| #2 | (high reliability NEXT organiz*) | 2 | 2 |
| #3 | (mindful NEXT organiz*) | 0 | 0 |
| #4 | #1 OR #2 OR #3 | 2 | 2 |
| #5 | high reliability OR "HRO" | 277 | 269 |
| #6 | MeSH descriptor: [Oncology Service, Hospital] explode all trees | 27 | 27 |
| #7 | MeSH descriptor: [Health Facilities] explode all trees | 23,372 | 23,163 |
| #8 | health care | 90,922 | 88,185 |
| #9 | #6 OR #7 OR #8 | 109,073 | 106,233 |
| #10 | oncology OR cancer | 254,455 | 250,789 |
| #11 | hospital OR "care unit" OR organization OR facilit* OR institution OR department | 717,867 | 704,308 |
| #12 | #10 AND #11 | 88,865 | 85,441 |
| #13 | #9 OR #12 | 191,120 | 185,713 |
| #14 | #5 AND #13 | 31 | 26 |
| #15 | #4 OR #14 | 32 | 27 |
| #16 | MeSH descriptor: [Neoplasms] explode all trees | 127,004 | 126,100 |
| #17 | oncology OR cancer OR Tumor OR Malignan* | 290,001 | 285,489 |
| #18 | #16 OR #17 | 308,275 | 303,729 |
| #19 | #15 AND #18 | 11 | 9 |
|  |  |  |  |
|  | **43(CINAHL) + 439(PubMe) +9(Cochrane) exclude duplicates** | **451** |  |
