# Supplementary material for: Enhancing High Reliability in Oncology Care: The Critical Role of Nurses—A Systematic Review and Thematic Analysis
Source: Healthcare (Basel). 2025 Jan 31;13(3):283. doi: 10.3390/healthcare13030283 (PMC11817837; doi:10.3390/healthcare13030283)
Supplement: Supplementary file 1 [file healthcare-13-00283-s001.zip › supplementary.file1 250112.docx]

| **Supplementary File 1 Search terms** | | | | | | | | | | |  |
| --- | --- | --- | --- | --- | --- | --- | --- | --- | --- | --- | --- |
| **Key terms** | **#1 High Reliability Organizations** | **#2Health care, (oncology) Health Facilities (a)** | **(b1)** | | **(b2)** | | **#3 Nurse’s Role（Nurse）** | | **#4 Oncology** | |  |
| PubMed/ MeSH | High Reliability Organizations | |  | |  | | Oncology Nursing | | Neoplasms | |  |
|  |  | Oncology Service, Hospital | |  | |  | | Nurse's Role | |  | |
|  |  | Health Facilities |  | |  | |  | |  | |  |
| PubMed/ Free Keywords | "high reliability organiz*" | |  | |  | | Nurses | | oncology | |  |
|  | "mindful organiz*" | |  | |  | | Health Personnel | | cancer | |  |
|  | "high reliability" | "health care" |  | |  | | "role of nurse" | | Tumor | |  |
|  | "HRO" |  |  | |  | | "Nursing Role" | | Malignan* | |  |
|  |  |  | oncology or cancer | | hospital | | "Professional Role" | |  | |  |
|  |  |  |  | | "care unit" | | "Medical Personnel" | |  | |  |
|  |  |  |  | | organization | | "Health Care Provider" | |  | |  |
|  |  |  |  | | facilit* | | "Healthcare Worker" | |  | |  |
|  |  |  |  | | institution | | "Health Care Professional" | |  | |  |
|  |  |  |  | | department | | "Health Care Staff" | |  | |  |

| **Key terms** | **#1 High Reliability Organizations** | **#2Health care, (oncology) Health Facilities (a)** | **(b1)** | **(b2)** | **#3 Nurse’s Role（Nurse）** | **#4 Oncology** |
| --- | --- | --- | --- | --- | --- | --- |
| CINAHL/ Hedings |  | Oncology Care Units |  |  | Oncology Nursing | Neoplasms+ |
|  |  | Cancer Care Facilities |  |  | Nursing Role |  |
|  |  | Health Facilities |  |  | Professional Role+ |  |
|  |  |  |  |  | Nurses+ |  |
|  |  |  |  |  | Health Personnel+ |  |
| CINAHL/  Free Keywords | "high reliability organiz*" | |  |  | role of nurse | oncology |
|  | "mindful organiz*" | |  |  | Nurse's Role | cancer |
|  | "high reliability" | "health care" |  |  | Health Care Staff | Tumor |
|  | "HRO" |  | oncology or cancer | hospital | Medical Personnel | Malignan* |
|  |  |  |  | "care unit" | Health Care Provider |  |
|  |  |  |  | organization | Healthcare Worker |  |
|  |  |  |  | facilit* | Health Care Professional |  |
|  |  |  |  | institution |  |  |
|  |  |  |  | department |  |  |
| **Key terms** | **#1 High Reliability Organizations** | **#2Health care, (oncology) Health Facilities (a)** | **(b1)** | **(b2)** | **#3 Nurse’s Role（Nurse）** | **#4 Oncology** |
| Cochrane Trials/ MeSH | High Reliability Organizations | |  |  | Oncology Nursing |  |
|  |  | Oncology Service, Hospital |  |  | Nurse's Role | Neoplasms |
|  |  | Health Facilities |  |  |  |  |
| Cochrane Trials/ Free Keywords | "high reliability organiz*" | |  |  | Nurses | oncology |
|  | "mindful organiz*" | |  |  | Health Personnel | cancer |
|  | "high reliability" | "health care" |  |  | "role of nurse" | Tumor |
|  | "HRO" |  |  |  | "Nursing Role" | Malignan* |
|  |  |  | oncology or cancer | hospital | "Professional Role" |  |
|  |  |  |  | "care unit" | "Medical Personnel" |  |
|  |  |  |  | organization | "Health Care Provider" |  |
|  |  |  |  | facilit* | "Healthcare Worker" |  |
|  |  |  |  | institution | "Health Care Professional" |  |
|  |  |  |  | department | "Health Care Staff" |  |
